# Efficacy and safety of a modified DVD regimen followed by lenalidomide for the treatment of Newly Diagnosed Multiple Myeloma

**DOI:** 10.1016/j.clinsp.2025.100575

**Published:** 2025-01-26

**Authors:** Zhichao Li, Wenhao Zhang, Fang Huang, Siguo Hao

**Affiliations:** a Department of Hematology, Xin Hua Hospital Affiliated to Shanghai Jiao Tong University School of Medicine, Shanghai, PR China; b Department of Lymphoma, Fudan University Shanghai Cancer Center, Shanghai, PR China

**Keywords:** Newly Diagnosed Multiple Myeloma, Pegylated Liposomal Doxorubicin, Bortezomib, Lenalidomide, Transplantation

## Abstract

**Background:**

The common drugs used for the treatment of Newly Diagnosed Multiple Myeloma (NDMM) include bortezomib and lenalidomide, but the adverse effects of lenalidomide cannot be ignored, especially when it is used in the initial therapy.

**Methods:**

This retrospective study evaluated the efficacy and safety of a modified DVD regimen (pegylated liposomal doxorubicin, bortezomib, and dexamethasone) followed by lenalidomide in the treatment of NDMM. A total of 40 NDMM patients were treated with a reduced dose of pegylated liposomal doxorubicin (20 mg/m^2^) on day 1, subcutaneous bortezomib (1.3 mg/m^2^) on days 1, 4, 8, and 11, and dexamethasone (20 mg) on days 1, 2, 3, 4, 8, 9, 11, and 12 (20 days for each course). When patients failed to achieve partial or better response after 2 courses of treatment, a regimen containing lenalidomide was administered. After the induction therapy, 15 eligible patients received Peripheral Blood Stem Cells (PBSC) mobilization and transplantation followed by maintenance therapy with lenalidomide.

**Results:**

The response rate (≥ very good partial response) was 55% and 80% after 2 and 4 courses, respectively. The 18-month Progression Free Survival (PFS) and Overall Survival (OS) were 78.6% and 83.4%, respectively. Grade 3 or 4 hematologic toxicity occurred in less than 10% of patients. In addition, all 15 transplant-eligible patients successfully mobilized PBSC (median CD34^+^cells = 4.59 × 10^6^/kg) and underwent autologous PSBC transplantation.

**Conclusions:**

This study suggests that the modified DVD regimen followed by lenalidomide is an effective and well-tolerated regimen, and has little influence on the PBSC collection and transplantation for patients with NDMM.

## Highlights

• Adverse effects of lenalidomide for Newly Diagnosed Multiple Myeloma (NDMM) cannot be ignored.
• Lower-dose PLD administered with dexamethasone and subcutaneous bortezomib is associated with a good durable response.
• Lenalidomide is retained as maintenance or salvage therapy if the DVD regimen fails.

## Introduction

Multiple Myeloma (MM) is a neoplasm of plasma cells characterized by overproduction of monoclonal proteins in the serum and/or urine, resulting in end organ dysfunctions, including bone lesions, renal damage, hypercalcemia, and/or anemia.^1^ With the emergence of drugs with new actions, such as Proteasome Inhibitors (PIs), Immunomodulatory Drugs (IMiDs), and monoclonal antibodies, the survival of MM patients has improved remarkably.^2^^,^^3^ In general, clinical studies have confirmed the effectiveness of these new drugs, and currently, a chemotherapy regimen is often composed of at least one new drug and a conventional chemotherapeutic agent (e.g., alkylating agents, anthracyclines, and glucocorticosteroids).^4^, ^5^, ^6^, ^7^

Lenalidomide, an immunomodulatory drug, is an integral part of current treatment paradigms for Newly Diagnosed Multiple Myeloma (NDMM), which has been supported by multiple clinical trials.^8^, ^9^, ^10^ However, lenalidomide-associated Adverse Events (AEs) need to be recognized, especially when it is used in the initial therapy.^11^ Neutropenia is the most common and serious AE after lenalidomide treatment. In the FIRST trial,^12^ grade 3/4 neutropenia was reported in 30% of patients receiving first-line continuous Rd (lenalidomide and dexamethasone). Notably, neutropenia occurs most frequently during the first six months of treatment, with incidence declining over time.^13^ A similar situation is present in the lenalidomide-associated thromboembolism which occurs most frequently in first-line treatment and rarely occurs in maintenance therapy with lenalidomide following ASCT.^14^^,^^15^ In the FIRST trial, despite thromboprophylaxis with aspirin, low-molecular-weight heparin, or warfarin, 5% of patients treated with continuous Rd still developed deep-vein thrombosis, and 4% experienced pulmonary embolism.^12^ As lenalidomide is excreted from the kidney, dose adjustment is required for MM patients with renal impairment, aiming to reduce the risk of developing side effects.^16^ Furthermore, it has been reported that up to 15% of MM patients may develop amyloidosis.^17^ Among amyloidosis patients, the deterioration of kidney function frequently occurs during the lenalidomide treatment.^18^ In the present study, lenalidomide treatment was postponed to maintenance therapy and a lenalidomide-based regimen was used as a salvage therapy.

Pegylated Liposomal Doxorubicin (PLD) is an improved formulation of standard doxorubicin, with reduced toxicity and improved pharmacokinetic properties. Given that PLD undergoes hepatic metabolism and is predominantly excreted in the bile, it does not increase the burden on the kidneys.^19^ Therefore PLD may be especially suitable for the treatment of patients with impaired renal function. Studies have shown that up to 50% of NDMM patients have renal impairment, 20% have severe acute kidney injury, and 5% require dialysis.^20^

Bortezomib, which revolutionizes the treatment of MM, has been used as a critical drug in most current anti-myeloma chemotherapy regimens. Bortezomib and PLD with or without Dexamethasone (DVD) have been used to treat MM patients. In a phase 3 randomized controlled clinical study, relapsed or refractory MM patients who were not previously treated with bortezomib were enrolled, 646 patients were randomly assigned to receive either Intravenous (IV) bortezomib (1.3 mg/m^2^) on days 1, 4, 8, and 11 (21 days for each course) or the same bortezomib regimen with PLD (30 mg/m^2^) on day 4.^7^ The combination regimen was significantly more effective than the single-agent regimen, showing a longer time to progression (9.3 months vs. 6.5 months, p = 0.000004), better complete or very good partial response rate (27% vs. 19%, p = 0.0157), and increased 15-month survival rate (76% vs. 65%, p = 0.03). However, compared with the single-agent regimen, the combined regimen was associated with increased toxicity: 80% of patients suffered grade 3/4 Adverse Events (AEs), resulting in 36% of patients discontinuing the treatment with a combination regimen. In the frontline setting, PLD (30 mg/m^2^) on day 4 was evaluated in combination with bortezomib,^21^ or PI and dexamethasone.^22^ Both studies suggest that patients have a higher response rate to regimens containing PLD, but as compared to doxorubicin, PLD fails to reduce the incidence of AEs, including neutropenia, thrombocytopenia, and hand-foot syndrome.

As the median age at diagnosis of MM patients is 69 years^23^ and many patients have comorbidities, balancing the efficacy and tolerability is key to the treatment of these patients. Therefore, some clinicians have adjusted the dosage of drugs and the schedule of drug administration. In a phase 2 prospective clinical study involving previously untreated MM patients, the dose of intravenous bortezomib was decreased from 1.3 mg/m^2^ to 1 mg/m^2^ on days 1, 4, 8, and 11 (4 weeks for each course), and results showed the incidence of Peripheral Neuropathy (PN) reduced, and both dexamethasone (40 mg) and PLD (5 mg/m^2^) were also administered on the same day as bortezomib was administered.^24^ The modified regimen achieved an Overall Response Rate (ORR) of up to 86% with a low incidence of AEs. The incidence of grade 3 neutropenia, thrombocytopenia, and PN was 5.7%, 2.9%, and 5.7%, respectively, and no grade 4 AEs occurred. As the incidence of PN was substantially reduced after subcutaneous bortezomib treatment as compared to the intravenous bortezomib treatment,^25^ it is unnecessary to reduce the initial dose of bortezomib. In this retrospective study, reduced-dose PLD (20 mg/m^2^, instead of 30 mg/m^2^) was administered on day 1 (3 weeks for a course), and bortezomib (1.3 mg/m^2^) was administered subcutaneously on days 1, 4, 8, and 11 combined with intravenous or oral dexamethasone (20 mg) on days 1, 2, 4, 5, 8, 9, 11, and 12. Except for the initial treatment, which was administered in the hospital, the rest of this regimen was completed in outpatient clinics.

## Study design

This study conforms to the STROBE statement and was approved by the Ethics Committee of Xin Hua Hospital, Affiliated to Shanghai Jiao Tong University School of Medicine (XHEC-D-2021–160), and conducted in compliance with the principles of the Declaration of Helsinki, the Code of Ethics of the World Medical Association. All the participants were fully informed before the study. Written informed consent was obtained from all the participants prior to study commencement. In this retrospective study, the medical records of 40 consecutive MM patients who received this modified DVD regimen followed by lenalidomide were reviewed. Eligible patients were those with previously untreated symptomatic MM and measured disease (defined as a monoclonal immunoglobulin spike of ≥ 5 g/L on serum electrophoresis or urine monoclonal immunoglobulin spike of ≥ 200 mg/24h or involved free light chain > 100 mg/L and abnormal serum free kappa-to-lambda ratio). The institutional review board approved this study before the collection of clinical information. Patients were excluded from this study if they had POEMS syndrome (plasma cell dyscrasia with polyneuropathy, organomegaly, endocrinopathy, monoclonal protein, and skin manifestations), plasma cell leukemia, primary amyloidosis, solitary plasmacytoma, or monoclonal gammopathy of undetermined significance.

All of the patients received treatment for at least two courses (21 days for each course) between July 8, 2017 and November 1, 2022. Data were collected up to November 30, 2022. Patients were treated with IV or oral dexamethasone (20 mg) on days 1, 2, 4, 5, 8, 9, 11, and 12, subcutaneous bortezomib (1.3 mg/m^2^) on days 1, 4, 8, and 11, and intravenous PLD (20 mg/m^2^) on day 1. The regimen was modified if patients did not achieve at least Partial Response (PR) after two cycles. Otherwise, patients proceeded with this regimen for four courses, and the appropriate patients were recommended to receive stem cell mobilization and Autologous Stem Cell Transplantation (ASCT). All of the transplantation-eligible patients received steady mobilization with Granulocyte Colony-Stimulating Factor (G-CSF) alone. High-dose melphalan (200 mg/m^2^) was used as the conditioning regimen for ASCT. For patients over 65 years or with renal impairment, the dose of melphalan was adjusted to 140 mg/m^2^. After ASCT, patients received lenalidomide-based maintenance therapy. Patients who did not undergo ASCT received up to six courses of DVD followed by lenalidomide-based maintenance therapy.

Clinical information was collected from the medical records of each patient at baseline and during the treatment. Data obtained included age, gender, International Staging System (ISS), Revised-ISS (R-ISS), MM subtype, and therapeutic response according to the International Myeloma Working Group (IMWG) criteria. Other laboratory data collected included complete blood count, serum albumin, serum creatinine, serum calcium, lactate dehydrogenase, β2-microglobulin, Free Light Chain (FLC), and cytogenetic information after standard banding method and fluorescence in situ hybridization. An echocardiography, skeletal survey, and bone marrow biopsy were done at baseline and repeated according to the symptoms and signs. Physical and neurological examinations were also performed at baseline and just before the start of each new treatment course. For patients receiving ASCT, the primary data included the collection yield of CD34^+^ cells and days of neutrophil and platelet engraftment.

The primary endpoints were the efficacy and safety of the DVD regimen in treating NDMM patients. Efficacy was determined based on ORR (Complete Response [CR], Very Good Partial Response [VGPR], PR, and Minimal Response [MR]) and the median time to best response. The efficacy of the DVD regimen was assessed in the third week of each course. Safety was evaluated according to the frequency and intensity of AEs and graded based on the National Cancer Institute Common Terminology Criteria for Adverse Events version 5.0. Secondary endpoints included Progression-Free Survival (PFS) and Overall Survival (OS). PFS was defined as the duration from the initiation of treatment to Progressive Disease (PD) or death of any cause, whichever occurred first. OS was defined as the interval between the initiation of therapy and the death of any cause.

Descriptive statistics of categorical and continuous data were generated, such as the mean, median, standard deviation, and range. The response rates were summarized with proportions. The PFS and OS were estimated with the Kaplan-Meier method, and group comparisons were performed with a log-rank test. A value of p < 0.05 was considered statistically significant. Statistical analyses were done using SAS version 9.2 (SAS Institute Inc., Cary, NC, USA).

## Results

### Characteristics of patients

A total of 40 consecutive patients with NDMM who had received DVD therapy in the hospital met the inclusion criteria. All the patients were treated between July 8, 2017 and November 1, 2022, and data were collected until November 30, 2022. The median duration of follow-up was 22 months (range 3–64 months). Before November 1, 2022, all the patients discontinued DVD treatment because of the following reasons: 20 completed treatments for 6 courses followed by maintenance therapy, 15 completed treatments for 4 courses followed by ASCT and maintenance therapy, 3 had the regimens adjusted in order to achieve better response, 1 had AEs, and 1 developed lung cancer. Demographic and clinical characteristics are summarized in Table 1.

**Table 1:** Demographic and baseline clinical characteristics (n = 40).

Characteristics	n	%
Age, median (range), years	63 (41‒72)	
Patients with age ≥ 65 years	14	35.0
Male	22	55.0
M-protein isotype		
IgG	16	40.0
IgA	10	25.0
Light chain only	14	35.0
ISS stage		
Ⅰ	8	20.0
Ⅱ	4	10.0
Ⅲ	28	70.0
R-ISS stage		
Ⅰ	7	17.5
Ⅱ	18	45.0
Ⅲ	15	37.5
Creatinine level, median (range), μmoL/L	113 (38‒715)	
Patients with creatinine > 177 μmoL/L	14	35.0
LDH, median (range), µ/L	173.5 (84‒912)	
Patients with LDH > ULN	12	30.0
Hemoglobin level, median (range), g/dL	81 (41‒134)	
Patients with hemoglobin < 10 g/dL	30	75.0
Calcium level, median(range), mmoL/L	2.38 (1.71‒3.68)	
Patients with calcium > 2.75 mmoL/L	12	30.0
Cytogenetics by FISH		
Standard-risk^a^	27	67.5
High-risk^a^	13	32.5

ISS, International Staging System; R-ISS, Revised-ISS; LDH, Lactate Dehydrogenase; ULN, Upper Limit of Normal; FISH, Fluorescence In Situ Hybridization.

a Standard-risk: No high-risk chromosomal abnormality. High-risk: Presence of del(17p), translocation (4;14) and/or translocation (14;16).

### Efficacy

The efficacy was evaluated in all the patients receiving the DVD regimen. The median number of courses completed was 4 (range 2–6). All the patients completed at least 2 courses of DVD treatment, and the response rate (≥ VGPR) after two courses was 55%. After two courses, 3 patients who did not achieve at least PR and 1 patient who developed grade 3 PN with pain had their regimens modified, and 1 patient discontinued treatment for the presence of lung cancer. The remaining 35 patients completed at least 4 courses of DVD treatment. The responses were achieved rapidly, the median time to response (at least PR) was 1 cycle (range 1 to 2 cycles) and the median time to best response was 2 courses (range 1 to 4 cycles). The response was evaluated throughout 4 courses of DVD induction and results showed the VGPR rates increased over the induction courses. Table 2, Table 3 outline the types of responses.

**Table 2:** Responses after 1 to 4 courses of DVD therapy, n (%).

Response	1 course (n = 40)	2 courses (n = 40)	3 courses (n = 35)	4 courses (n = 35)
sCR	0	0	1 (2.9)	7 (20.0)
CR	4 (10.0)	7 (17.5)	7 (20.0)	7 (20.0)
VGPR	4 (10.0)	15 (37.5)	16 (45.7)	14 (40.0)
PR	21 (52.5)	15 (37.5)	11 (31.4)	7 (20.0)
MR	7 (17.5)	2 (5.0)	0	0
≥VGPR	8 (20.0)	22 (55.0)	24 (68.6)	28 (80.0)
≥PR	29 (72.5)	37 (92.5)	35 (100.0)	35 (100.0)

sCR, stringent Complete Response; CR, Complete Response; VGPR, Very Good Partial Response; PR, Partial Response; MR, Minimal Response.

**Table 3:** Response rate (≥ PR) after 2 courses based on baseline clinical variables.

Patients with different variables	Patients achieving ≥ PR after 2 courses (n)		p
	Yes	No	
< 65 years old	24	2	0.9498
≥ 65 years old	13	1	
ISS Ⅰ/Ⅱ stage	11	1	1.0000
ISS Ⅲ stage	26	2	
R-ISSⅠ/Ⅱ stage	24	1	0.2779
R-ISS Ⅲ stage	13	2	
Creatinine ≤177 μmoL/L	24	2	0.9498
Creatinine >177 μmoL/L	13	1	
LDH ≤ ULN	24	2	0.9498
LDH > ULN	13	1	
Hemoglobin < 10 g/dL	28	2	1.0000
Hemoglobin ≥ 10 g/dL	9	1	
Calcium ≤ 2.75 mmoL/L	27	1	0.2093
Calcium > 2.75 mmoL/L	10	2	
High-risk cytogenetics	12	1	1.0000
Standard-risk cytogenetics	25	2	

PR, Partial Response; ISS, International Staging System; R-ISS, Revised-ISS; LDH, Lactate Dehydrogenase; ULN, Upper Limit of Normal.

**Fig. 1:**
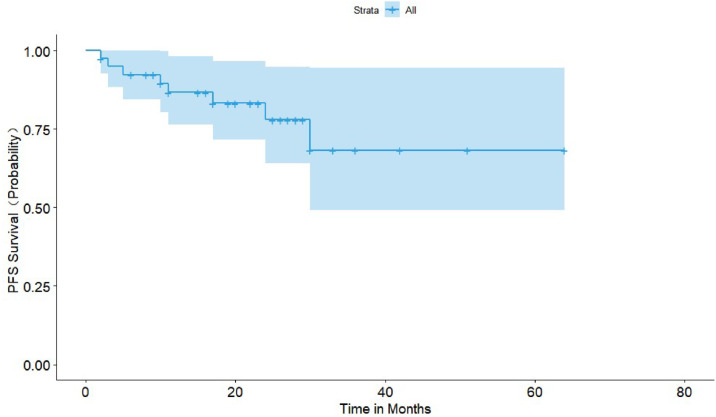
Kaplan-Meier estimate of Progression-Free Survival (PFS).

**Fig. 2:**
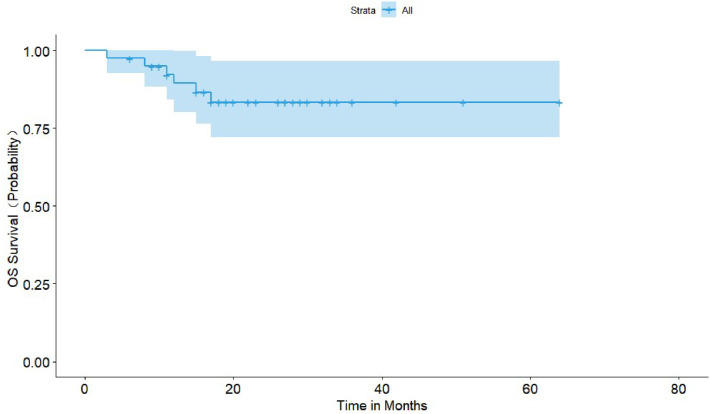
Kaplan-Meier estimate of Overall Survival (OS).

Before November 30, 2022, seven patients died, six of whom died of PD, and one died of lung cancer. Thirty-three patients remained alive, three of whom suffered from disease relapse. The median duration of follow-up was 22 months (range 3–64 months), and the median PFS and OS were not reached. The 18-month PFS and OS were 78.6% (95% CI 65.9% to 91.3%) and 83.4% (95% CI 71.9% to 94.9%), respectively (Fig. 1, Fig. 2).

After 4 courses of induction therapy, 15 transplantation-eligible patients received Peripheral Blood Stem C (PBSC) mobilization with G-CSF alone. An adequate amount of CD34^+^ cells was collected in all the patients (median = 4.59 × 10^6^/kg, range, 2.51‒10.22 × 10^6^/kg). The median cycle of apheresis was 2 (range 1‒3). All these patients underwent ASCT, either single (n = 14) or double (n = 1). Neutrophil and platelet counts recovered at a median of 10 (range 8‒12) and 12 (range 9‒14) days respectively after the first or PBSC infusion.

### Safety and tolerability

AEs are listed in Table 4. Results showed the DVD regimen was well tolerated, and no treatment-related mortality was observed. Overall, AEs were manageable.

**Table 4:** Incidence and severity of adverse events.

Advent events (n = 40)	All grades (%)	Grade 3 (%)	Grade 4 (%)
Hematologic			
Neutropenia	21 (52.5)	3 (7.5)	0
Thrombocytopenia	17 (42.5)	2 (5)	1 (2.5)
Anemia	6 (15)	2 (5)	0
Nonhematologic			
Pneumonia	12 (30)	1 (2.5)	0
Peripheral neuropathy	10 (25)	1 (2.5)	0
Elevation of transaminase	9 (22.5)	1 (2.5)	0
Diarrhea	8 (20)	1 (2.5)	0
Constipation	7 (17.5)	2 (5)	0
Upper respiratory infection	4 (10)	0	0
Urinay tract infection	3 (7.5)	0	0
Abdominal pain	3 (7.5)	0	0
Hand-foot syndrome	2 (5)	0	0
Vomiting	2 (5)	0	0
Shingles	1 (2.5)	0	0
Headache	1 (2.5)	0	0
Rash	1 (2.5)	0	0
Deep vein thrombosis	1 (2.5)	0	0
Cholecystitis	1 (2.5)	1 (2.5)	0
Hypertension	1 (2.5)	0	0
Loss of appetite	1 (2.5)	0	0
Epileptiform seizures	1 (2.5)	0	0
Fatigue	1 (2.5)	0	0
Somnolence	1 (2.5)	0	0
Hypotension	1 (2.5)	1 (2.5)	0

Hematologic toxicities were generally mild to moderate. No grade 4 anemia or neutropenia occurred, and one patient suffered grade 4 thrombocytopenia. The most common nonhematologic AE was pneumonia, which occurred in 12 patients (30%), and only 1 patient (2.5%) experienced grade 3 pneumonia. The second most common nonhematologic event was PN which occurred in 10 patients (25%), and only 1 patient (2.5%) developed grade 3 PN. Notably, this AE was reversible in most cases. Other common AEs included elevation of transaminase (22.5%), diarrhea (20%), constipation (17.5%), and upper respiratory infection (10%). Importantly, only 2 patients had grade 1/2 Hand-Foot Syndrome (HFS).

## Discussion

In this retrospective study, the DVD regimen was evaluated in patients with NDMM. Lenalidomide was postponed as maintenance therapy or salvage therapy if the DVD induction failed. Patients achieved a very high response rate to this modified regimen using dexamethasone and subcutaneous bortezomib, while lower dose PLD showed a marked reduction in the toxicity profile as compared to other similar regimens. In an open-label phase 2 trial,^22^ NDMM patients were treated with bortezomib (1.3 mg/m^2^) on days 1, 4, 8, and 11, PLD (30 mg/m^2^) on day 4, and oral dexamethasone, the ORR (≥ PR) was 85.0% (CR/near-CR 37.5%; VGPR or better 57.5%), and 1-year PFS was 92.5%. However, this regimen was also associated with more AEs. Neutropenia and thrombocytopenia occurred in 50% and 90% of patients, respectively. PN was found in 92.5% of patients, and HFS in 75% of patients. In the present study, only 2 (5.9%) patients experienced HFS. 11 (32.4%) patients exhibited grade 1/2 PN, and only 1 patient showed grade 3 PN. Notably, PN resolved in 10 (91%) patients, which was in line with the results of the APEX phase 3 trial.^26^

In this study, lenalidomide treatment was postponed to maintenance therapy and a lenalidomide-based regimen was used as the salvage therapy if DVD induction failed. Three patients who did not achieve ≥ PR after 2 courses of DVD treatment received treatment with a VRD regimen. Two of them achieved PR, and the other patient suffered disease progression and died 6 months later.

ASCT has been proven to be a preferred treatment for MM.^27^ Successful mobilization and collection of PBSC are essential for ASCT. However, current mobilization strategies often have a failure rate of as high as 5%‒25% among patients with MM.^28^^,^^29^ The mobilization failure depends on many factors including the type of induction therapy.^30^ In this study, the DVD regimen was used as induction therapy and the use of lenalidomide was postponed because it may impair PBSC collection.^29^ All the transplantation-eligible patients had an adequate number of mobilized PBSCs with G-CSF alone and underwent ASCT successfully.

In conclusion, this retrospective study shows that lower-dose PLD (20 mg/m^2^) administered with dexamethasone and subcutaneous bortezomib is associated with a high rate of durable response and improved tolerability for patients with newly diagnosed MM. Lenalidomide is retained as maintenance or salvage therapy if the DVD regimen fails. Additionally, this regimen seems to have little influence on the PBSC collection and engraftment. More prospective trials are required to confirm the efficacy and safety of this modified DVD regimen followed by lenalidomide in MM patients.

## Ethics approval and consent to participate

This study was approved by the Ethics Committee of Xin Hua Hospital Affiliated to Shanghai Jiao Tong University School of Medicine (XHEC-D-2021–160), and conducted in compliance with the principles of the Declaration of Helsinki, the Code of Ethics of the World Medical Association. All the participants were fully informed, and written informed consent was obtained from all the participants.

## Ethics approval and consent to participate

Not applicable.

## Consent for publication

Not applicable.

## Authors’ contributions

Hao Siguo and Huang Fang conceived the study, critically reviewed the intellectual content of the manuscript and made substantive revisions to the important contents of the manuscript. Li Zhichao and Zhang Wenhao were the major contributors to the research and the writing of the manuscript.

## Funding

Not applicable.

## Declaration of competing interest

The authors declare no conflicts of interest.
